# Global gene expression analyses of hematopoietic stem cell-like cell lines with inducible *Lhx2 *expression

**DOI:** 10.1186/1471-2164-7-75

**Published:** 2006-04-06

**Authors:** Karin Richter, Valtteri Wirta, Lina Dahl, Sara Bruce, Joakim Lundeberg, Leif Carlsson, Cecilia Williams

**Affiliations:** 1 Umeå Center for Molecular Medicin, Umeå Universitet, 90187 Umeå, Sweden; 2 School of Biotechnology, KTH, Royal Institute of Technology, AlbaNova University Center, 10691 Stockholm, Sweden; 3 Department of Biosciences at Novum, Karolinska Institutet, 14157 Huddinge, Sweden

## Abstract

**Background:**

Expression of the LIM-homeobox gene *Lhx2 *in murine hematopoietic cells allows for the generation of hematopoietic stem cell (HSC)-like cell lines. To address the molecular basis of *Lhx2 *function, we generated HSC-like cell lines where *Lhx2 *expression is regulated by a tet-on system and hence dependent on the presence of doxycyclin (dox). These cell lines efficiently down-regulate *Lhx2 *expression upon dox withdrawal leading to a rapid differentiation into various myeloid cell types.

**Results:**

Global gene expression of these cell lines cultured in dox was compared to different time points after dox withdrawal using microarray technology. We identified 267 differentially expressed genes. The majority of the genes overlapping with HSC-specific databases were those down-regulated after turning off *Lhx2 *expression and a majority of the genes overlapping with those defined as late progenitor-specific genes were the up-regulated genes, suggesting that these cell lines represent a relevant model system for normal HSCs also at the level of global gene expression. Moreover, *in situ *hybridisations of several genes down-regulated after dox withdrawal showed overlapping expression patterns with *Lhx2 *in various tissues during embryonic development.

**Conclusion:**

Global gene expression analysis of HSC-like cell lines with inducible Lhx2 expression has identified genes putatively linked to self-renewal / differentiation of HSCs, and function of *Lhx2 *in organ development and stem / progenitor cells of non-hematopoietic origin.

## Background

A small number of hematopoietic stem cells (HSCs) are responsible for the continuous production of mature blood cells throughout life. This process is based on the capability of the HSC to replenish itself through a process called self-renewal [1-3], and to differentiate into all hematopoietic lineages. Consequently, analysis of the mechanisms underlying HSC self-renewal and differentiation is fundamental for understanding the maintenance of the normal hematopoietic system. At present, our knowledge of these processes on the molecular and cellular level is limited, since studies on HSCs are hampered by their low abundance in hematopoietic organs and are thus difficult to access in sufficiently large quantities for direct studies. An increase in the number of HSCs occurs under normal physiological conditions in the liver during embryonic development [4], indicating that the microenvironment in the fetal liver efficiently promotes self-renewal of HSCs. Elucidation of the mechanisms responsible for the expansion of the hematopoietic system during embryonic development might therefore offer insights into the mechanisms of self-renewal in the hematopoietic system.

The expansion of the hematopoietic system is intimately connected with the development of the liver, suggesting over-lapping molecular mechanisms of these processes. Liver development in the mouse is initiated at embryonic day 8 (E8) when a distinct region of the ventral foregut endoderm receives inductive signals from two adjacent tissues, the septum transversum mesenchyme and the pre-cardiac mesoderm [reviewed in [5]]. Ventral foregut endodermal cells committed to hepatic fate proliferate and form a liver bud from which hepatoblasts migrate and intermingle with cells of the septum transversum mesenchyme. The mesenchymal cells originating from the septum transversum thereby contribute to the mesenchymal part of the liver, and development into a functional organ relies on continuous interactions between the mesenchymal and endodermal portions of the liver [6-8]. At E10 the liver has become a distinct organ with discernible lobes and is infiltrated by numerous hematopoietic cells and cells with HSC properties can be detected in the liver by E11 [9].

Members of the LIM-homeodomain transcription factor family play critical roles during embryonic development in both vertebrates and invertebrates by controlling processes such as asymmetric cell division, tissue specification and differentiation of specific cell types [reviewed in [10]]. One member of this family, *Lhx2*, is of particular interest, based on its function in the development of several different tissues via mesenchymal-epithelial interactions and regulation of stem/progenitor cells [11-17]. *Lhx2 *is expressed in the liver-associated septum transversum mesenchyme that becomes an integral part of the liver and its expression is maintained during liver development until adult stage in hepatic stellate cells [12,16]. *Lhx2*^-/- ^embryos display a decreased size of the liver manifested already at E10.5, suggesting that *Lhx2 *is required for expansion of the fetal liver [12,17]. The mutant phenotype is due to the presence of activated hepatic stellate cells causing a fibrotic and disorganized liver containing phenotypically abnormal endodermal cells [12,16]. The mesenchymal defect in the liver of *Lhx2*^-/- ^mice cause a lethal anemia, which is cell non-autonomous since the *Lhx2*^-/- ^hematopoietic cells appears to be normal [17], suggesting that the mutant microenvironment is unable to support hematopoietic development. These observations indicate that *Lhx2 *expression in hepatic stellate cells is involved in mesenchymal-epithelial cell interactions important for liver expansion, organization, differentiation and formation of the hematopoietic microenvironment in the fetal liver.

To further elucidate the putative role of Lhx2 in the hematopoietic system we ectopically expressed Lhx2 in hematopoietic progenitor/stem cells derived from embryonic stem (ES) cells differentiated *in vitro *and from adult bone marrow (BM) cells. This approach allowed for the generation of immortalized multipotent and Steel factor-dependent hematopoietic progenitor cell (HPC) lines [18,19]. The HPC lines share several characteristics with normal HSCs such as response to specific cytokines/growth factors, expression of transcription factors and interactions with stromal cells [18,20]. The pattern of cell surface markers expressed by HPC lines derived from ES cells and adult BM is similar to that of early fetal and adult HSCs, respectively. The HPC lines derived from adult BM can generate erythroid, myeloid and lymphoid cells following transplantation into lethally irradiated recipients, and can long-term engraft stem cell-deficient mice [19]. The cells engrafting the stem cell-deficient mice maintain high level of expression of *Lhx2 in vivo*, which eventually leads to a chronic myeloproliferative disorder resembling human chronic myeloid leukemia [21]. Thus, ectopic Lhx2 expression in hematopoietic cells allows for the generation of HSC-like cell lines, and molecular analyses of these HSC-like cell lines would give information of the role of Lhx2-induced self-renewal of HSCs, and hence novel insights into stem cell physiology and pathology.

In order to elucidate the molecular basis of *Lhx2*-induced self-renewal of HSCs we generated HSC-like cell lines from ES cells differentiated *in vitro *with *Lhx2 *expression controlled by a tetracycline-responsive element, and hence dependent on the presence of the tetracycline-analogue doxycyclin (dox) in the culture media [22]. These dox-dependent hematopoietic progenitor cell (DoxHPC) lines down-regulate *Lhx2 *expression almost two orders of magnitude within 24 hrs after dox withdrawal, leading to rapid differentiation into various myeloid cell types. We used the DoxHPC lines to analyse *Lhx2 *function by comparing global gene expression in the presence of dox to different time points after dox withdrawal using cDNA array technology. This approach identified 267 genes differentially expressed at all time points, and thus putatively involved in *Lhx2 *function in stem cell self-renewal and / or differentiation, and during organ development.

## Results

### Generation of HPC lines from the ES cells with inducible *Lhx2 *expression

**Figure 1:**
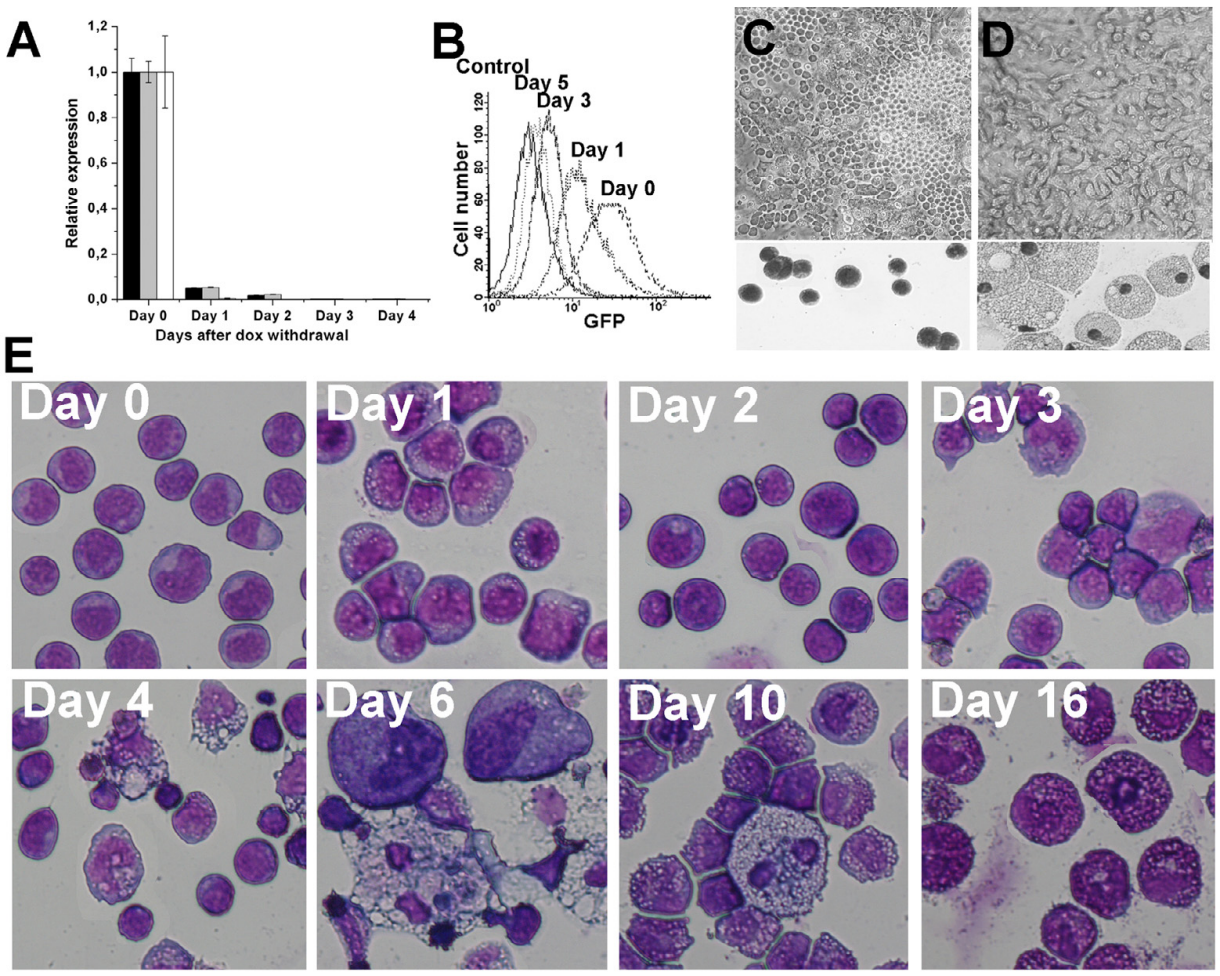
**Characterization of the DoxHPC lines. A. **Real-time PCR analyses of *Lhx2 *expression in three independent DoxHPC lines at the indicated time points after dox withdrawal. Relative *Lhx2 *expression is compared to the expression in each DoxHPC lines in the presence of dox (Day 0) and this expression is arbitrarily set as 1. **B. **Flow cytometry analysis of GFP expression at the indicated time points after dox withdrawal in the DoxHPC line where GFP expression is linked to *Lhx2 *expression by an ires. **C. **Cobblestone areas formed after seeding DoxHPC lines onto S17 stromal cells (upper panel) and maintenance of blast-like cells in these cultures (lower panel) in the presence of dox. **D. **Lack of cobblestone areas after seeding DoxHPC lines onto S17 stromal cells (upper panel) and differentiation into macrophages (lower panel) in the absence of dox. **E. **May-Grünwald Giemsa staining of cytospun cells from a DoxHPC line cultured in the presence of dox (Day 0) as compared to different time points after dox withdrawal.

Previously, when generating HPC lines, we used ES cells transduced with retroviral vectors containing *Lhx2 *cDNA that were subsequently differentiated *in vitro *into embryoid bodies (EBs). The HPC lines were established by expanding progenitor cells expressing *Lhx2 *present in colonies showing a distinct morphology that appeared in the clonal assays of EB cells [18,23]. A similar approach was used here, however, since we were unable to identify distinct colonies in the clonal assays containing dox (probably due to the efficient up-regulation of *Lhx2 *expression in all cells), 96 colonies generated in the factor combination supplemented with dox were randomly picked and expanded individually in medium containing SF, IL-6 and dox. Stable doxycyclin-dependent hematopoietic progenitor cell (DoxHPC) lines could be established from 66 (69%) of these 96 colonies. The pattern of cell surface marker expression was similar to that previously reported for the HPC lines generated from day 6 EBs [18,23], e.g. c-kit^+^/CD45^+^/CD41^+^/Sca-1^-^/Lin^- ^(data not shown). All DoxHPC lines expressed *Lhx2 *and transcription of *Lhx2 *was efficiently down-regulated (>90%) within 24 hours after dox withdrawal and was undetectable after 72 hours (Figure 1A). In the DoxHPC7 line where GFP was linked to *Lhx2 *expression, flow cytometry analyses revealed similar instantaneous and efficient down-regulation of GFP expression after dox withdrawal (Figure 1B). Rapid cellular changes appeared immediately after dox withdrawal with respect to differentiation status and fraction of apoptotic cells. The fraction of apoptotic cells increased from a background level of 5% (cells in the presence of dox) to a maximum of 35% five days after dox withdrawal. The fraction of apoptotic cells decreased thereafter to background level within ten days after dox withdrawal (data not shown). Under these culture conditions (SF+IL-6 without dox), differentiation into various myeloid cells such as neutrophilic granulocytes, macrophages and megakaryocytes, could usually be observed after 4–5 days (Figure 1E), with concomitant up-regulation of lineage specific cell surface markers (data not shown). Although the distribution of the various myeloid cells differed between different DoxHPC lines after dox withdrawal, all DoxHPC lines maintained in SF+IL-6 without dox generated mast cells and beyond eight days this cell type dominated the cultures (Figure 1E). Immature hematopoietic progenitor cells can functionally interact with stromal cells *in vitro *by generating cobblestone areas [24] in long-term bone marrow culture) and are referred to as cobblestone area forming cells (CAFCs) [25]. We have previously shown that the HPC lines derived from ES cells differentiated *in vitro *efficiently generated cobblestone areas when seeded onto stromal cells [20]. The DoxHPC lines only formed cobblestone areas when seeded onto stromal cells in the presence of dox (Figure 1C), whereas rapid differentiation into macrophages occurred in the absence of dox (Figure 1D). Thus, this is a reproducible and efficient system amenable for global gene expression analyses comparing *Lhx2*^+ ^stem cells to their immediate *Lhx2*^- ^progeny.

### Analysis of gene expression changes caused by down-regulation of *Lhx2*

**Figure 2:**
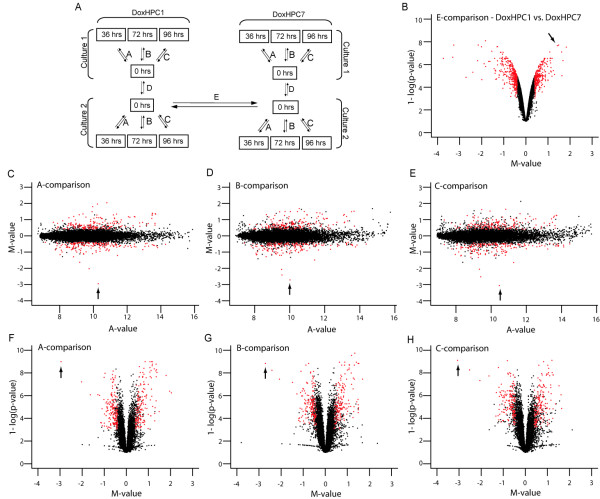
**Overview of the microarray expression data. A. **Design of the experiment. This study includes 30 hybridizations using cDNA arrays (ArrayExpress accession numbers A-MEXP-175 and E-MEXP-431). Fourteen slides were allocated for profiling each of the DoxHPC1 and DoxHPC7 lines. Technical replicates were included at three different levels (replicated cell cultures of both cell lines, replicated hybridizations in each comparison using reciprocal dye assignments and repeated measurements using probes printed twice on each array). The hybridization scheme also includes two hybridizations between the two cultures within each cell line, as well as two hybridizations between the different *Lhx2*-expressing cell lines. The 36, 72 and 96 hours refer to the time after *Lhx2 *expression was down-regulated through withdrawal of dox from the culture medium. Each arrow denotes one hybridization. The tip and the base of the arrow indicate Cy5 and Cy3 assignments of the dyes, respectively. Letter assignments by the arrows (A, B, C and D) are used to describe the comparisons in the text. Figure **C, D **and **E **show the MA-plots for the comparisons at 36 (A), 72 (B) and 96 hours (C) respectively. Figure **B, F-H **show the statistical significance (1 - log of p-value) for each probe to be differentially expressed versus the M-value in a volcano-shaped plot; **B **between the cell lines DoxHPC1 and DoxHPC7 in E-comparison at time 0 hours and **F-H **corresponding values for the A-C comparison. Differentially expressed genes are shown in red. The feature corresponding to *Lhx2 *is indicated with an arrow.

We used cDNA microarray technology to investigate the genome-wide transcriptional effects of down-regulation of *Lhx2 *expression in the DoxHPC1 and DoxHPC7 lines. Gene expression at 36, 72 and 96 hours after dox withdrawal were analysed to elucidate differential gene expression early after *Lhx2 *down-regulation, but prior to the time point when the majority of the cells are committed myeloid cells. The hybridizations were carried out using the design depicted in Figure 2A. The number of features that remained on each array after filtering was high (82–92%) and the majority of the excluded features were removed by the first filtering step (i.e. GenePix and manual flagging).

**Figure 3:**
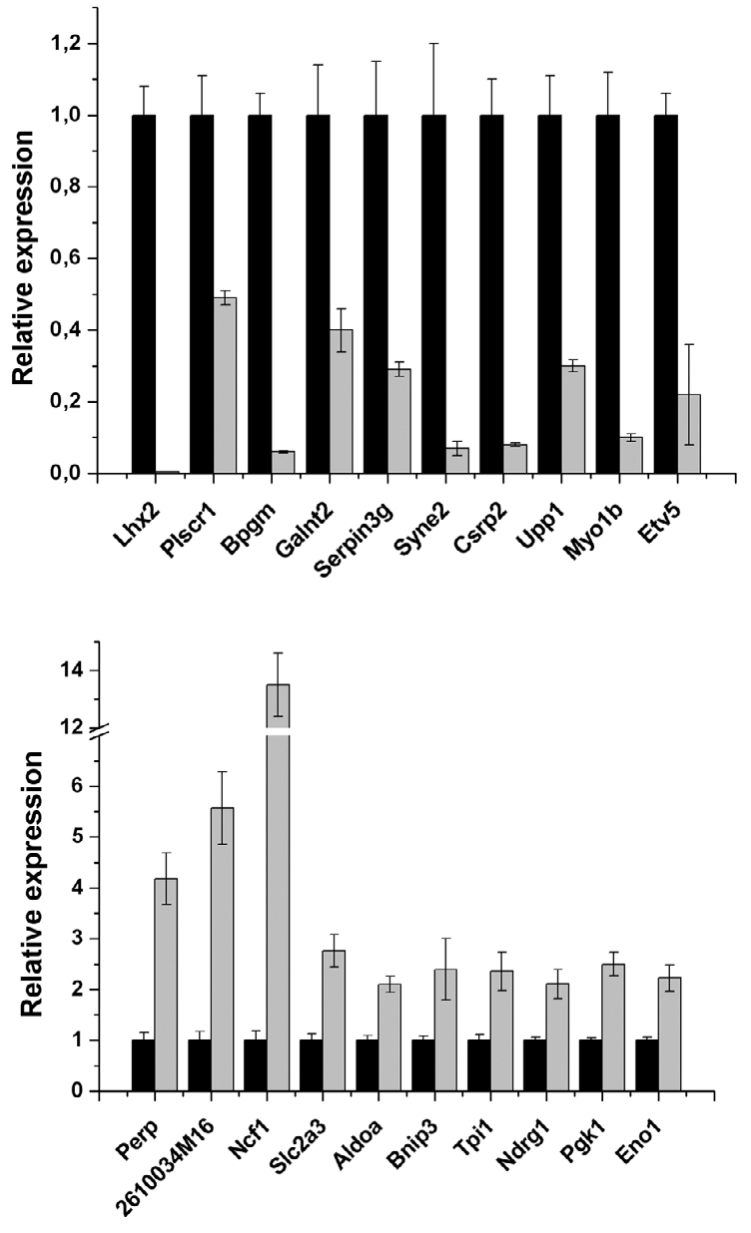
**Real-time PCR analyses confirm the cDNA array data**. Expression analyses of 10 genes (including *Lhx2*) with down-regulated expression when *Lhx2 *expression is turned off (upper panel) and 10 genes with up-regulated expression when *Lhx2 *expression is turned off (lower panel). Relative expression of each gene is set as 1 when *Lhx2 *is expressed (Day 0) and is compared to the expression at day 3 after dox withdrawal.

**Figure 4:**
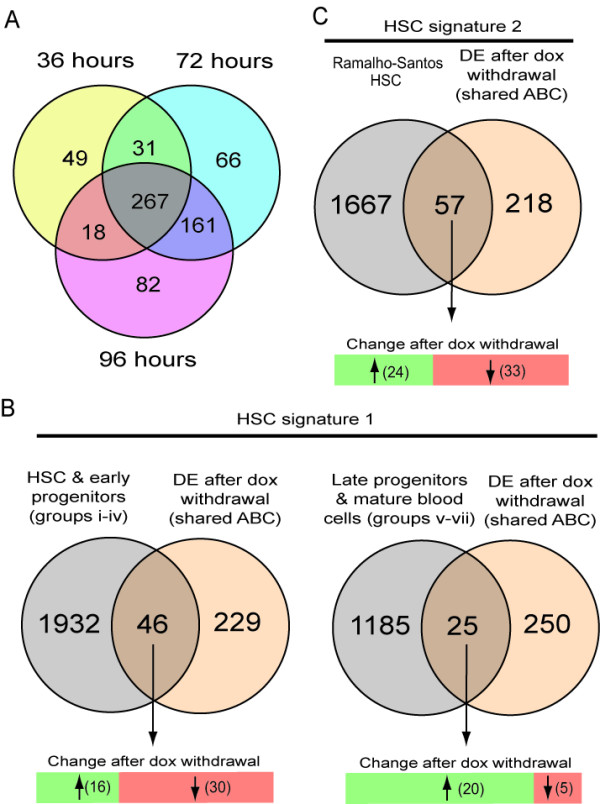
**Comparisons between previously published stem cell signatures. A.**Our study. Overlap between differentially expressed genes at 36, 72 and 96 hours after *Lhx2 *expression was down-regulated. The intersections between groups of differentially expressed genes are visualized using Venn-diagrams (for clarity not drawn to proportions). The numbers refer to unique genes. **B-C. **Comparison between the transcripts differentially expressed in our study and two previously published hematopoietic stem cell-enriched gene expression signatures. Signature 1 (B) is derived from the [30] study and Signature 2 (C) from the [29] study. Transcripts that are differentially expressed in our study at all three time-points are included. The overlapping transcripts are further split into transcripts that are up- and down-regulated in our study. Comparison between our data set and the two HSC signatures was carried out on the UniGene transcript level (UniGene build 147).

We used an empirical Bayes moderated t-test to identify differential gene expression. Genes with a false-discovery rate adjusted p-value lower than 0.01 were considered differentially expressed. However, several of these showed small fold-changes, and therefore only genes with an absolute M-value higher than 0.4 (corresponds to a 1.3-fold difference) were included in further analysis. The results are summarized in Table 1, 2 and 3, and Figure 2 and 4A. Complete results are available as supplementary data. Comparison of the two independent cultures within each *Lhx2*-expressing cell line at time-point 0 (D-comparisons) indicated no differential expression (data not shown). On the other hand, the comparison between DoxHPC1 and DoxHPC7 lines (E-comparison) indicated that a large number of genes differed in their expression levels (Figure 2B). In total, 646 differentially expressed array probes were identified (see Additional file 2), which could be further divided into 265 probes (206 genes) with higher expression in DoxHPC1 and 381 probes (295 genes) with higher expression in DoxHPC7. Interestingly, the expression of *Lhx2 *is 2.7-fold higher in DoxHPC1 as compared to DoxHPC7, which may at least partly explain the difference between the cell lines. To identify genes differentially expressed as a consequence of *Lhx2 *down-regulation, we combined the data for DoxHPC1 and DoxHPC7 cell lines. This analysis revealed 463 probes (365 genes) at 36 hours, 695 probes (525 genes) at 72 hours and 699 probes (528 genes) at 96 hours to be differentially expressed (Figure 2C–H). A large proportion of these genes were differentially expressed at all three time-points (Figure 4A and Additional file 1). This shared set of 267 genes included 141 down-regulated and 126 up-regulated genes. The array data confirms the dox-dependent regulation of *Lhx2*, as *Lhx2 *is the gene with the largest decrease in expression (down by ~90%) after dox withdrawal (indicated by the arrows in Figure 2B–H). Agreement in the M and p-values was observed in cases where several independent probes represented different regions of the same gene (data not shown), further validating the accuracy of the method. Differential expression of 10 down-regulated and 10 up-regulated selected genes was confirmed by using real-time PCR to compare gene expression in the DoxHPC1 line in the presence of dox to three days after dox withdrawal (Figure 3).

Twenty-six of the 337 probes that were differentially expressed at all three time points were not included in UniGene build 144. We used Blast sequence similarity searches [26] to annotate the corresponding ESTs and the results are provided in the (see Additional file 1). Several of the unknowns map to either 5' or 3' UTRs, but also partly to known genes such as *Wbscr5*, *Map3k7*, and *Eifay/Eifax*. Also, one of the probes [GenBank:CX207886] has no matches in any public sequence repository. Whether these transcripts correspond to novel genes or novel splice variants of already defined genes remains to be determined.

### Gene Ontology functional analysis of differentially expressed genes

We used the Gene Ontology classification of the 267 differentially expressed genes for a two-step functional analysis to define the overall Gene Ontology category representation and to identify themes that were over-represented compared to the complete mouse transcriptome [27]. A Gene Ontology annotation was assigned to approximately 50% of the 141 down-regulated and 126 up-regulated genes. Within the 'Biological processes' branch of Gene Ontology, the largest groups that changed their expression upon *Lhx2 *down-regulation were 'macromolecule metabolism' (20 down-regulated / 42 up-regulated), 'signal transduction (19/20), 'cell growth and/or maintenance' (18/19), 'nucleobase, nucleoside, nucleotide and nucleic acid metabolism' (18/17) and 'biosynthesis' (8/20). A complete listing, which also includes the 'Molecular function' and 'Cellular component' branches of the Gene Ontology classification, is available as Supplementary data (see Additional file 3). The themes 'regulation of signal transduction', 'organogenesis' and 'cell death' were significantly over-represented among the down-regulated genes (i.e. self-renewal-specific) (Table 4). Several metabolism-related genes were significantly enriched among the up-regulated genes (i.e. differentiation-specific), which was also evident when the data was analysed using the KEGG metabolic pathways [28] (Table 4). Of note is that ten genes encoding components of the glycolytic pathway, including a glucose transporter (*Slc2a3*) and glycogen synthase, are among the genes up-regulated after dox withdrawal (see Additional file 1 and 3), suggesting that this metabolic pathway is important when stem cells are initiating differentiation.

### Comparison with HSC gene expression signatures

Global gene expression in murine HSCs has previously been studied using Affymetrix arrays [29,30]. Both studies analysed the transcriptome of HSCs by comparing gene expression in stem cell-enriched populations to that of more differentiated cells (late progenitors and Lin-positive mature blood cells in Ivanova *et al*., and the main cell population of the bone marrow in Ramalho-Santos *et al*.). We compared the genes enriched in these HSCs populations to our list of differentially expressed genes (at UniGene transcript level 275 UniGene clusters correlated to the previously defined 267 differentially expressed genes, where 144 Unigene clusters were down-regulated and 131 were upregulated). In the comparison with the data from Ivanova *et al*. (Signature 1 in Figure 4B), we divided their dataset into two groups; "HSC and early progenitor-enriched" (groups i-iv in Ivanova *et al*.) or "Late progenitor and mature blood cell-enriched" (groups v-vii). Of our differentially expressed Unigene transcripts 46 overlapped with those in the "HSC and early progenitor-enriched" transcripts and 25 overlapped with those in the "Late progenitor and mature blood cell-enriched" transcripts (Figure 4B). A majority (30 or 65%) of the 46 Unigene transcripts overlapping with the "HSC and early progenitor-enriched" signature were down-regulated after dox withdrawal, and a majority of the transcripts overlapping with the late progenitors (20 genes or 80%) were up-regulated after dox withdrawal. The comparison between the HSC-enriched transcripts from the study by Ramalho-Santos *et al*. (Signature 2 in Figure 4C) and our set of differentially expressed transcripts showed an overlap of 57 genes. Also in this comparison many transcripts (33, or 58%) were found to be down-regulated after dox withdrawal (Figure 4C). Thirteen genes were found in common between all three studies (Smo, Tes, Upp1, Laptm4b, Slco3a1, Fkbp1a, Itga6, A530057M15Rik, 6430596G11Rik, 1810009M01Rik, 1110004D19Rik, D13Ertd275e, and AA536749).

Collectively these results show that *Lhx2 *expression partly maintains the HSC signature and hence, the DoxHPC lines with inducible *Lhx2 *expression is a relevant model system for normal HSCs also at the level of global gene expression.

### Genes most affected by *Lhx2 *expression

The fifty genes that show largest fold-change (up- or down-regulated) after dox withdrawal are presented in Table 2 and 3. The list of down-regulated genes contains two members of the serpin family of protease inhibitors, *Serpina3g *and *Serpina3n*, where the former is the most highly differentially expressed gene. In general, serpins are involved in diverse processes such as coagulation, extra cellular matrix degradation, complement activation, fibrinolysis and apoptosis [31]. The expression pattern of Serpina3n has not been well characterized, but expression of *Serpina3g *is highly enriched in normal HSCs [32], and in the hematopoietic precursor cell line FDCP-Mix [33]. The exact biological function of *Serpina3g *is not known, but has been shown to involve inhibition of caspase-independent cell death [34]. This is in agreement with the observed increase in apoptosis after turning *Lhx2 *expression off, which is also reflected by the observation that two pro-apoptotic genes, *Bnip3 *and *Perp *[35,36], are among the 25 most up-regulated genes (Table 3). Over-expression of *Serpina3g *in FDCP-Mix cells caused delay in differentiation and increased clonogenic potential of these cells [33], suggesting similar function in the *Lhx2 *expressing HPC lines and normal HSCs.

Most genes encoding enzymes within the glycolytic pathway were up-regulated after *Lhx2 *expression was turned off, but one highly differentially expressed gene within this pathway, *Bpgm *(2,3-bisphosphoglycerate mutase), was an exception as it showed a decrease by 75%. This enzyme is known to be present in erythrocytes where it is involved in the reaction generating the metabolite 2,3-bisphosphoglycerate, which is important for the oxygen-hemoglobin interaction. However, *Bpgm *is also expressed during early developmental stages (egg and pre-implantation stages) [37], suggesting that it may affect other cellular processes.

Genes linked to both normal and abnormal hematopoietic progenitor cell function were identified among the top 50 most differentially expressed genes. For example, among the most down-regulated genes *Sox4 *and *Plscr1 *(Phospholipid scramblase 1), have both been reported to be enriched in HSCs [29,30]. Although the normal function of *Sox4 *in HSCs is not known, over-expression of *Sox4 *in mouse HSCs has recently been shown to cause myeloid leukemia and *Sox4 *is also a frequent insertional mutagenesis target in murine myeloid leukemias [38,39]. *Plscr1*-deficient mice have no major hematopoietic defects, but *Plscr1*^-/- ^hematopoietic progenitor cells show decreased colony formation and impaired differentiation to neutrophils in response to Steel factor and G-CSF [40]. Examples of the most up-regulated genes were *Ndrg1 *(N-myc downstream regulated gene 1), and *Ncf1 *(Neutrophil cytosolic factor 1). Up-regulation of these genes would be expected since the DoxHPC lines under the culture conditions used herein differentiate towards both neutrophil and mast cell lineages, and these genes are linked to the development and/or function of these cell types [41,42].

*Etv5 *and *Csrp2 *(cysteine and glycine-rich protein 2), which also showed significant decrease in expression after turning *Lhx2 *expression off, have been implicated in mesenchyme-epithelia interactions during embryogenesis [43,44], similar to *Lhx2*. Interestingly, both *Csrp2 *and *Lhx2 *are specifically expressed in hepatic stellate cells of the liver [12,45]. Chronic liver injury causes activation of hepatic stellate cells leading to hepatic fibrosis [46], and both *Lhx2 *and *Csrp2 *have been negatively implicated in this process [16,45], suggesting overlapping function of these genes also in non-hematopoietic cells. In this respect it is interesting to note that one of the top 25 up-regulated genes is P4ha1 (proline-4-hydroxylase), which encodes an enzyme important for post-translational modifications of collagen, leading to increased stability of collagen fibrils. Expression of P4ha1 is also up-regulated in the fibrotic *Lhx2*^-/- ^fetal liver as compared to the normal fetal liver [16]. Thus, analyses of differentially expressed genes identified in this hematopoietic-based system might also give insights into *Lhx2 *function (and lack of function) in non-hematopoietic cells.

### Overlapping gene expression patterns in mouse embryos of *Lhx2 *and genes down-regulated in DoxHPC lines upon dox removal

**Figure 5:**
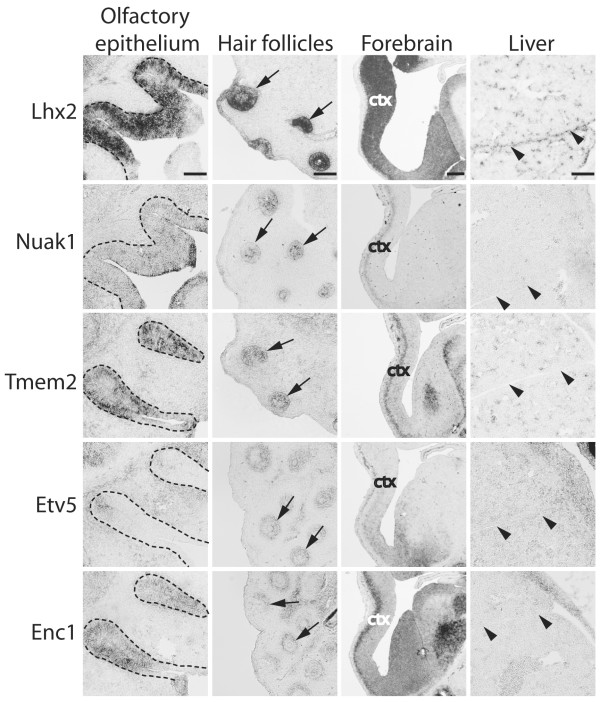
**Gene expression analyses in mouse embryos of genes with down-regulated expression when *Lhx2 *expression is turned off. ***In situ *hybridization using probes of the indicated genes on sagital sections on E12.5-13.5 embryos of the indicated tissues. The border between the olfactory epithelium and the underlying mesenchyme is outlined in each section by a broken line. Examples of individual hair (whisker) follicles are indicated by arrows in each section. The part of the developing forebrain that gives rise to the cerebral cortex is indicated by ctx in each section. Arrowheads indicate the border between individual liver lobes in each section. Scale bars sections of Olfactory epithelium, Hair follicles and Liver; 100 μm, Fore brain; 200 μm.

*Lhx2 *plays an important role during embryonic development in the formation of several different tissues via mesenchymal-epithelial interactions and/or regulation of stem/progenitor cells [11-17]. We therefore wanted to elucidate whether any of the differentially expressed genes, particularly genes that were down-regulated upon dox withdrawal, also were expressed in tissues/organs where *Lhx2 *is expressed during embryonic development. Although we were comparing differential expression in hematopoietic cells, 6 of 13 (46 %) of the genes down-regulated after dox withdrawal that we have analysed thus far, *Nuak1*, *Tmem2*, *Etv5*, *Enc1*, *Csrp2*, and *Tgfb1i1 *(Transforming growth factor β1 induced transcript 4), showed partly over-lapping expression pattern with *Lhx2 *in different tissues. *Lhx2 *is expressed by cells in the liver, the hair follicles, the developing cerebral cortex of the forebrain and the olfactory epithelium, and examples of genes showing overlapping gene expression with *Lhx2 *in two or more of these tissues are shown in Figure 5. *Tmem2 *is expressed in all four tissues, *Nuak1 *and *Enc1 *are expressed in olfactory epithelium, hair follicles, cortex area of the forebrain but not within the liver lobes, whereas *Etv5 *is expressed hair follicles, cortex area of the forebrain, weakly within the liver lobes but not in olfactory epithelium (Figure 5). These results suggest that the mechanism whereby *Lhx2 *immortalizes hematopoietic stem/progenitor cells might partly overlap with the function of *Lhx2 *in the development of a variety of organs.

## Discussion

We have generated HSC-like cell lines by differentiating ES cells with inducible *Lhx2 *expression *in vitro*. Self-renewal of these DoxHPC lines, is strictly dependent on *Lhx2 *expression, since down-regulation of *Lhx2 *expression leads to loss of CAFCs and rapid and efficient differentiation into a variety of myeloid cells. To elucidate the putative function of *Lhx2 *in stem cells, we used microarray technology to compare global gene expression in these cell lines in the presence of dox (*Lhx2 *expression on) to different time points after dox withdrawal (*Lhx2 *expression off). We identified 267 genes (141 down-regulated and 126 up-regulated) that showed differential expression at all time points (36, 72 and 96 hours) after *Lhx2 *expression was turned off. Gene Ontology classification revealed that genes related to 'regulation of signal transduction', 'organogenesis' and 'cell death' were over-represented among the down-regulated genes (i.e. "stem cell-specific"), and that metabolism-related genes were over-represented among the up-regulated genes (i.e. "differentiation specific").

We have previously shown that the HPC lines generated by *Lhx2 *expression resemble normal HSCs in many basic molecular, cellular and biochemical aspects [18-20,23,47]. Moreover, the HPC lines derived from bone marrow that engrafted stem cell-deficient mice maintained *Lhx2 *expression *in vivo *and caused myeloproliferation [21]. We therefore anticipated identifying genes involved in both physiological and pathological processes affecting HSCs by analyses of global gene expression of the DoxHPC lines. A comparison of the differentially expressed genes to previously defined HSC gene expression signatures revealed that a majority of the overlapping genes expressed in HSC-containing populations were those down-regulated after *Lhx2 *expression was turned off. Thus, although we have used a different microarray platform, the genes defined as stem cell-specific in our system (e.g. the 144 Unigene clusters that belonged to group of genes down-regulated after dox withdrawal), reveal a 21 and 23 % overlap with genes defined as HSC-specific in the two different stem cell databases, respectively (see Additional file 4). Furthermore, thirteen of the 144 Unigene clusters (9 %) defined as stem cell-specific in our study overlapped with both HSC databases (see Additional file 4). In interpreting the results from the comparison between our stem cell-specific genes and the HSC-enriched genes in Ivanova *et al*. [30] and Ramalho-Santos *et al*. [29], it should be noted that the probes on the arrays in those studies only has an overlap in our array of 50 and 57%, respectively. The overlap from our model system for HSCs could be further viewed in relation to a study showing that the overlap between three different HSC-specific datasets varied between 19 and 32 % [48]. It should be noted that, despite the fact that these studies were carried out using the same Affymetrix GeneChips, identical analysis approaches and essentially the same cell populations, there is still considerable variability between the different HSC-specific datasets. Moreover, a majority of the genes that were up-regulated after *Lhx2 *expression was turned off were preferentially expressed in differentiated cell populations. These results suggest that there is molecular overlap in the self-renewal and differentiation process between the DoxHPC lines and normal HSCs also at global gene expression level. We could also identify genes linked to malignant transformation of hematopoietic cells and one such example is Sox4, which was recently shown to cause acute leukemia if over-expressed in HSCs [38]. Sox4 has also been identified as a frequent insertional mutagenesis target in murine myeloid leukemias [39], further supporting a role of this gene in malignant transformation. Since *Lhx2 *is always integrated into the same position, close to the *Hprt1 *gene in the DoxHPC lines, expression of insertional mutagenesis target genes is not due to insertional mutation, as has been suggested for retroviral integration [49], hence, these genes are more likely to be linked to *Lhx2 *function and/or stem cell function. Human chronic myeloid leukemia cells have been reported to express *Lhx2 *although its function in the disease is not clear [50]. Another gene down-regulated after *Lhx2 *expression is turned off is *Prkd2 *(Protein kinase D2, see Additional file 1), which has also been shown to be a substrate for the BCR-ABL fusion protein present in chronic myeloid leukemia cells [51]. These results reveal a putative functional link between *Lhx2 *and the BCR-ABL fusion protein. However, both *Sox4 *and *Prkd2 *have been shown to be preferentially expressed in HSCs [29,30], which suggests overlapping molecular mechanism regulating both HSC physiology and pathology. By using our inducible system it would be possible to dissect these processes on the molecular, cellular and biochemical level.

It has been suggested that the Notch pathway and pathways activated by different morphogens such as Wnt, Bone morphogenetic proteins (BMP) and hedgehog, are important regulators of stem cell function [52-55]. We have previously shown that the *Lhx2*-induced HPC lines self-renew by a cell nonautonomous mechanism [20], and the anemia in *Lhx2*^-/- ^mouse embryos is due to a cell nonautonomous mechanism, suggesting that *Lhx2 *regulates genes encoding proteins involved in cell-cell interactions [17]. However, we have not found any evidence for differential expression of major mediators of the Notch or Wnt pathways in the DoxHPC lines after turning *Lhx2 *expression off. Although the microarrays we used do not contain all mediators of these pathways, the results suggest that the mechanism whereby *Lhx2 *expression generates HPC lines is partly or completely independent of at least the Wnt and Notch pathways. We did find consistent down-regulated expression of the hedgehog receptor *Smo *(Smoothened) after *Lhx2 *expression was turned off (see Additional file 1), and *Smo *has also been shown to be preferentially expressed in normal HSCs (see Additional file 4) [29,30]. However, expression of the ligands (Sonic, Indian and Desert hedgehog) was very low to undetectable by real-time PCR analyses of the cell lines (data not shown). Thus, the functional role, if any, of hedgehog signalling in these cells remains to be determined. Furthermore, *Tsg *(Twisted gastrulation), which is a soluble modulator of BMP-signalling, was identified among the genes down-regulated after *Lhx2 *expression was turned off (see Additional file 1), and is also defined as an HSC-specific gene (see Additional file 4) [29,30]. The functional relevance of this observation remains to be determined as Tsg protein has been show to act as a BMP antagonist as well as a BMP agonist [56,57].

*Lhx2 *appears to play an important role during embryonic development in the formation of several different tissues via mesenchymal-epithelial interactions and/or regulation of stem/progenitor cells [11-17]. One of our aims was therefore to identify gene(s) directly or indirectly regulated by *Lhx2*, possibly providing insights into the function of *Lhx2 *in these processes. A caveat to this assumption is that it is not known whether *Lhx2 *functions as a transcriptional activator, repressor, or both. The only reported target genes putatively regulated by *Lhx2 *in a direct manner are those encoding olfactory receptors [58] and these receptors are not expressed in *Lhx2*^-/- ^olfactory epithelia [11,13]. These observations suggest that genes activated by *Lhx2 *are down-regulated in *Lhx2*^-/- ^animals, and conversely, genes repressed by *Lhx2 *are up-regulated in *Lhx2*^-/- ^animals. A putative example of the latter is *P4ha1 *as this gene was up-regulated after *Lhx2 *is turned off in DoxHPC lines, and the expression of this gene was also up-regulated in the liver of *Lhx2*^-/- ^embryos compared to wild type embryos [16]. Although our analysis was made in hematopoietic cells, almost 50% of the genes analysed among those down-regulated after dox withdrawal (*Lhx2 *expression turned off), showed similar expression pattern as *Lhx2 *in different non-hematopoietic organs/cells during embryonic development. Preliminary data suggests, however, that several of these genes show maintained expression pattern in *Lhx2*^-/- ^embryos, suggesting that these genes are not direct transcriptional targets for *Lhx2*. A plausible explanation for this observation is that different genes exert their function via parallel pathways in the regulation, formation or differentiation of a certain tissue but are not dependent on each other for their expression in that particular tissue. An illustrative example of such parallel but equally important pathways during organ formation can be observed during eye development, where both *Lhx2 *and *Pax6 *null mutant (Sey) embryos show a developmental arrest at the optic vesicle stage [17,59]. However, the expression of these genes appear to be independent of each other since *Pax6 *isexpressed in the arrested optic vesicle in the *Lhx2*^-/- ^embryos and *Lhx2 *is expressed in the arrested optic vesicle in *Pax6*^-/- ^embryos [17]. Whether the genes identified in this study play an equally important role as *Lhx2 *in the development of the respective organ remains to be determined. Our approach may therefore have identified both genes that are directly or indirectly regulated by *Lhx2*, and genes that play an important role together with *Lhx2 *via parallel pathways but are independent of *Lhx2 *expression, similar to the *Lhx2*/*Pax6 *example during eye formation. These results suggest that elucidation of *Lhx2 *function in various organs would give further insights into *Lhx2 *function in the DoxHPC lines and hence both physiological and pathological processes regulating normal HSCs. Conversely, elucidation of *Lhx2 *function in DoxHPC would give insights into *Lhx2 *function in various basic processes during embryonic development, such as mesenchymal-epithelial interactions and regulation of stem/progenitor cells in non-hematopoietic tissues. We are at present time exploring all alternatives of *Lhx2 *function presented above.

## Conclusion

Expression of the LIM-homeobox gene *Lhx2 *in hematopoietic cells derived from both ES cells differentiated in vitro and from bone marrow, allows for the generation of HSC-like cell lines. To address the molecular basis of *Lhx2 *function, we have analysed global gene expression in HSC-like cell lines with inducible Lhx2 expression. This approach identified 267 differentially expressed genes where 141 (144 Unigene clusters) were down-regulated and 126 (131 Unigene clusters) were up-regulated when Lhx2 expression was turned off. The relevance of this model system for normal HSC function was revealed by that the majority of the differentially expressed genes overlapping with HSC-specific datasets were defined as stem cell-specific in our system, and the majority of the differentially expressed genes overlapping with more differentiated hematopoietic cell population were defined as differentiation- or commitment-specific in our system. Moreover, gene expression analyses in various tissues during embryonic development of the down-regulated genes revealed that almost half of these genes showed overlapping expression patterns with *Lhx2*, suggesting a functional link to the development and regulation of various tissues and stem/progenitor cells. Thus, this approach has identified genes putatively linked to self-renewal / differentiation of HSCs, and function of *Lhx2 *in organ development and stem / progenitor cells of non-hematopoietic origin.

## Methods

### Generation of ES cells with inducible *Lhx2 *expression

The ES cell line Ainv15 was maintained on irradiated mouse embryonic feeder (MEF) cells in Dulbecco's modified Eagle medium (DMEM) (Gibco-BRL, United Kingdom) supplemented with 15% fetal calf serum (FCS) (Boehringer, Germany), 1.5 × 10^-4 ^M monothioglycerol (MTG) (Sigma, Germany) and leukaemia inhibitory factor (LIF) (Chemicon, Ca, USA). *Lhx2 *cDNA or *Lhx2 *cDNA linked to a green fluorescent protein cDNA preceded by an internal ribosomal entry site (*Lhx2*-ires-GFP), was inserted into the plox vector and transfected into the Ainv15 ES cells together with Cre recombinase cDNA as previously described [22], and subsequently cultured in 200 μg/ml G418 (Gibco-BRL). Clones of ES cells resistant to G418 were isolated, pooled and expanded.

### Differentiation of ES cell *in vitro*

ES cells used for *in vitro *differentiation were made feeder-independent in serum-free medium as previously described [60,61]. Briefly, ES cells cultured on MEF cells were trypsinized and transferred to gelatinized culture flasks in N2B27 medium (Gibco-BRL) supplemented with 10^4 ^U/ml LIF and 10 ng/ml BMP4 (R&D-systems, United Kingdom). The ES cells were trypsinized and transferred into Iscove's modified Dulbecco's media (IMDM) (Gibco-BRL) supplemented with 15% FCS (Integro Inc., The Netherlands), 4.5 × 10^-4 ^M MTG and 25 μg/ml ascorbic acid (Sigma) at 10^3 ^cells/ml. Embryoid bodies (EBs) were collected after five days of differentiation, resuspended in trypsin-EDTA and incubated for three minutes. Two ml of FCS was added and the cells were gently passaged through a syringe with a 20-gauge needle. Ten ml of IMDM medium was added; the cells were spun down and resuspended in fresh IMDM medium.

### Progenitor (clonal) assays of EB cells

The progenitor assays were carried out in IMDM containing 1% methylcellulose (Fluka, Switzerland) and supplemented with L-glutamine, 300 g/ml iron-saturated transferrin (Boehringer), 5% protein-free hybridoma medium II (Gibco-BRL), 10% plasma-derived serum (Antech Inc., Tx, USA), 100 ng/ml murine Steel factor (SF) R&D-systems), 10 ng/ml human IL-6 (R&D-systems), 4 IU/ml erythropoietin (Eprex Janssen-Cilag, Sweden) and with or without 2 μg/ml doxycyclin (dox) (Sigma). 10^5 ^or 2 × 10^5 ^EB cells were plated in triplicates in a final volume of 1.25 ml in 35-mm Petri dishes (Falcon 1008). Primitive erythroid colonies were scored after 4–6 days of incubation and definitive hematopoietic colonies (e.g. SF/IL-6-responsive) were scored after 10–12 days of incubation.

### Generation and maintenance of DoxHPC lines with *Lhx2 *expression regulated by the tet-on system

Individual colonies were randomly picked from the clonal assays after 12 days of incubation, transferred to 96-well plates and expanded in IMDM supplemented with 5% FCS, 1.5 × 10^-4 ^M MTG, 100 ng/ml SF, 10 ng/ml IL-6 and 2 μg/ml dox. Stable cell lines could be established from all cultures containing cells with blast-like morphology after three weeks in liquid culture. These cell lines were subsequently maintained in this media at cell densities between 5 × 10^5 ^and 2 × 10^6 ^cells/ml as previously described [20]. The efficiency of the system was verified by removing dox and analysing *Lhx2 *transcription by real-time PCR or GFP expression by flow cytometry at different time points after dox withdrawal (Figure 1A and 1B). No cell lines could be established from the control cell line expressing GFP alone or from colonies expanded in the absence of dox, as such cells differentiated into mast cells under these culture conditions. Two cell lines called DoxHPC1 and DoxHPC7 were selected for further gene expression analysis. The DoxHPC7 line was established from the ES cells where GFP was linked to *Lhx2 *expression.

### Design of the microarray experiment

The gene expression changes in DoxHPC1 and DoxHPC7 cell lines were analysed using the hybridization scheme in Figure 2A. Total RNA prepared from *Lhx2 *expressing cells was used as reference and compared to total RNA prepared from three time-points (36, 72 and 96 hours) after dox withdrawal. Two independent cell cultures were carried out for both DoxHPC1 and DoxHPC7 lines, providing a first level of replication. Each comparison was further analysed using two replicated hybridizations with the dye assignments reversed, providing a second level of replication. Finally, each probe was printed in duplicate on the arrays, which for each comparison results in eight measurements using three different levels of replication. Additional hybridizations carried out included replicated comparisons between the two separate cultures of each cell line, and a direct replicated comparison between *Lhx2*-expressing DoxHPC1 and DoxHPC7 cell lines.

### The cDNA microarray

The cDNA arrays were produced at the KTH Microarray Center and contain 14,121 in-house sequenced cDNA clones originating from a mouse brain lateral ventricle wall library, a normalized neurosphere library, an adult bone-marrow derived hematopoietic stem cell line expressing *Lhx2 *and additional control features including the Lucidea Universal ScoreCard probes (Amersham Biosciences, Sweden). A detailed description of the libraries is available elsewhere [62]. Details regarding the probes and cDNA amplification, purification and printing are available through the ArrayExpress microarray data repository using the array accession number A-MEXP-175. In brief, the cDNA inserts were amplified using Platinum *Taq *DNA polymerase (Invitrogen, Sweden), purified using the Multiscreen-PCR 384-well plates (Millipore, Sweden) automated on the Biorobot 8000 platform (Qiagen, Germany) and printed in either 30 or 50% DMSO. Probes were printed with a feature-to-feature distance of 175 μm into two identical fields consisting of 24 blocks each. The printed DNA was attached to the reactive surface of the Ultra-GAPS slides (Corning B.V., The Netherlands) using 250 mJ/cm^2 ^UV light. All cDNA sequences on the chip are deposited in the dbEST sequence database [63] and are accessible with the GenBank accession numbers listed in the ArrayExpress database. All clone annotations are derived from *Mus musculus *UniGene build 144, unless otherwise stated.

### Target preparation

Total RNA extraction from eight DoxHPC1 and eight DoxHPC7 samples was carried out using the RNeasy technology (Qiagen) and quality and quantity determined using the RNA Nano LabChip kit on the Agilent 2100 bioanalyzer (Agilent Technologies, Ca, USA) and Nanodrop ND-1000 spectrophotometer (Nanodrop Technologies, De, USA), respectively. For each cDNA synthesis reaction 20 μg of total RNA was mixed with 5 μg of random hexamer primer (Invitrogen), incubated at 70°C for 10 minutes, and finally on ice for at least 5 minutes. Reverse-transcription reaction mixture and 400 units of Superscript II RT-polymerase were added to yield a final volume of 30 μl containing first-strand buffer (Invitrogen), 10 mM DDT (Invitrogen) and 0.5 mM dNTPs (Sigma). The ratio of aminoallyl-modified dUTP to dTTP was 4:1 in the dNTP mixture. The reaction was carried out at 42°C for two hours, followed by 15 minutes hydrolysis of the RNA strand at 70°C in the presence of 16 mM EDTA (Sigma) and 150 mM NaOH. The reaction was neutralized using 150 mM HCl and purified using the MinElute Reaction Cleanup system (Qiagen) with the provided wash and elution buffers replaced by 80% ethanol and 100 mM NaHCO_3_, pH 9.0, respectively. A repeated elution from the column was carried out, generating a total volume of 20 μl. This was transferred to an aliquot containing one tenth of the monofunctional NHS-ester Cy3 or Cy5 dye tubes (Amersham Biotech), which had been dissolved in DMSO and subsequently dried in a vacuum centrifuge. After a 30-minute incubation in darkness at room temperature, the samples to be co-hybridized on a slide were pooled and purified using the MinElute columns.

### Microarray hybridization and signal extraction

To avoid unspecific hybridization to the surface the slides were pre-hybridized for 30 minutes in a solution consisting of 5xSSC, 0.1% SDS (Sigma) and 1% BSA (Sigma). Slides were subsequently washed in water and isopropanol (Sigma) and dried using a slide centrifuge. The labeled, pooled and denatured (3 minutes at 95°C) samples in a hybridization mixture containing 25 μg mouse Cot-1 DNA (Invitrogen), 40 μg poly-(dA) DNA (Operon),25% formamide (Sigma), 5xSSC and 0.1% SDS were applied under a LifterSlip cover (Erie Scientific Company, Nh, USA) and hybridized for 16–20 hours at 42°C in a water bath. After hybridisation the slides were washed with increasing stringency using 2xSSC and 0.1% SDS at 42°C, followed by 0.1xSSC and 0.1% SDS at room temperature and finally five times with 0.1xSSC at room temperature. Scanning was carried out at 10-μm resolution using the G2565BA DNA microarray scanner (Agilent Technologies) for which the photo multiplier tube was set to 100. The obtained TIFF-images were analysed using the GenePix Pro 5.1 software (Axon Instruments, Ca, USA). For each slide the foreground and background signal intensities were separated using the irregular feature-finding algorithm implemented in the software. A manual inspection was carried out to verify the results. The raw data, including several quality parameters, is available from the ArrayExpress data repository using the experiment accession number E-MEXP-431.

### Microarray data analysis

Data processing and identification of differentially expressed genes was carried out in the R environment for statistical computing and programming [64] using the Bioconductor package bundle [65], Limma [66], aroma package [67] and the kth-package [68]. As a first step the Cy3 and Cy5 intensities without background subtraction were converted to M (=log_2 _[red channel/green channel]) and A (=log_2 _[red channel*green channel]/2) format using the median intensity values for both channels. A feature was considered unreliable and removed from further analysis if GenePix flagged it as "Not Found" or if it was manually flagged as "Bad"during the image analysis step. Furthermore, if a feature satisfied one of the following criteria it was considered unreliable: a) both channels were saturated (above 65190 intensity units), b) the percentage of foreground pixels above the local background plus two of its standard deviations were below 70 for both channels of a feature, or c) the signal-to-noise ratio (defined as [mean foreground-mean background]/background standard deviation) for both channels was below 3. Remaining data was normalized using the intensity-dependent print-tip lowess method [69] and differentially expressed genes identified using the empirical Bayes moderated t-test implemented in the Limma package. As a first step the signal intensities for duplicate features on each array were averaged. Replicated hybridizations are expected to have a higher correlation than the repeated cell cultures for each cell line. This was considered in the linear model by first estimating the between-replicates correlation by using restricted maximum likelihood methods to estimate a common correlation for all probes [70]. Secondly, a linear model was fit for each probe using generalized least squares that takes into account the between-replicates correlation. In the third step an empirical Bayes approach was used to shrink the gene-wise standard errors towards a common value and a moderated t-statistic was calculated [66,71]. To compensate for multiple testing, the p-values associated with this t-statistics were adjusted using a false-discovery rate approach [72] implemented in R. Finally, probabilities for differential expression (B-values) were calculated for each gene. For these calculations the a priori assumption of differentially expressed genes was set to 0.01.

Classification into Gene Ontology functional groups [27] and analysis of over-represented themes was carried out using the EASE-package available at [73,74]. The complete mouse transcriptome was used for calculation of the expected frequencies in the over-representation analysis, and a Gene Ontology theme (detail level 3) was considered over-represented if the calculated EASE score was below 0.1.

### Analysis of differentially expressed genes by real-time PCR

Total RNA was extracted from cell pellets with TRizol reagent (Invitrogen). cDNA was synthesized by using the First-strand cDNA synthesis kit (Amersham Biosciences). Real-time PCR reactions were carried out in triplicates using SYBR green PCR master mix (Applied Biosystems, Ca, USA) and PCR products were detected with an ABI prism 7000 instrument (Applied Biosystems). The expression levels of the genes tested were normalized to the expression levels of Gapdh and confirmed with two additional housekeeping genes: Hprt1 (hypoxanthine phosphoribosyltransferase 1) and Tbp (TATA box binding protein) (data not shown). The following primers were used in the PCR analyses: *Lhx2 *Forward (F)- GCC GAG AAA GCG CAA GAG T and Reverse (R)- TGT TCA GCA TCG TTC TCG TTA CA; Plscr1 F- AGC TGC TGT TCC GAC ATT GA and R- GGA ACT GGA TCC CAA AAT TGT CT; Bpgm F- CTT AAA GGG CAA AAG CAT TCT GAT and R- TGG GCA GAG TGA TGT TGA TAA TAT C; Galnt2 F- TGC GGG TCC TCA GAA ATG A and R- TCA GCA ACC CGC TCC AA; Serpina3g F- CCT ACA GAT CCT GGC AGA GTT CA and R- GAT CTT CCC CTG GGT GTG ATT; Syne2 F- GGA GGT GTT CGG CAG AGT GT and R- TCT TCT ATG TCC GTC TCA TTC TCA GA; Csrp2 F- CCG TGT ATG CTG CGG AGA A and R- TTG GCA CAC CGG AAA CAG T; Upp1 F-TGA CCG CTA CGC CAT GTA TAA A and R- CAT GAT GCC GAT GGA AGG A; MyoIb F- ATC AGG TCA AGG AAC AGC TTC TG and R- TCA TTC CTC ACA GTC TTG GCA TT; Etv5 F- GAG CCG CTC TCT CCG CTA T and R- CCG GGT CAC ACA CAA ATT TG; Perp F- CTT GTT TTC CTG AGA GTC ATT GGA and R- GGT TAT CGT GAA GCC TGA AGG T; 2610034M16Rik F- GAG GAC TCA CTC AGG TTT TGT GAA and R- AAT GTC AAC TTC TGC TCC TTC TAA TTT TA; Ncf1 F- CAA AGA TGG CAA GAA TAA CGT AGC T and R- AGT CAG CAA TGG CCC GAT AG; Slc2a3 F- ACG ATC GGC TCT TTC CAG TTT and R- TTC TAA CCG CTC TTC CAA AGT GTA; Aldoa F-CCC TTC CCC CAA GTT ATC AAG and R- GGC ACC ACA CCC TTA TCT ACC T; Bnip3 F- GGT TTT CCT TCC ATC TCT GTT ACT GT and R- GTT GTC AGA CGC CTT CCA ATG; Tpi1 F- ACC GAG AAG GTC GTG TTC GA and R- GGC CAG GAC CAC CTT GCT; Ndrg1 F- CAT CGG CAT GAA CCA CAA GA and R- AAA ATG TTG TGT GAT CTC CTG CAT; Pgk1 F- GGA AGC GGG TCG TGA TGA and R- GCC TTG ATC CTT TGG TTG TTT G; Eno1 F- GGC ACC CTC TTT CCT TGC TT and R- GGC GTG GAT CCT GAG AAT AGA C; Gapdh F- CGT GTT CCT ACC CCC AAT GT and R- TGT CAT CAT ACT TGG CAG GTT TCT; Hprt F- GCA GTA CAG CCC CAA AAT GG and R- AAC AAA GTC TGG CCT GTA TCC AA; Tbp F- GAA TTG TAC CGC AGC TTC AAA A and R- AGT GCA ATG GTC TTT AGG TCA AGT T.

### *In situ *hybridizations

Embryos were fixed in 4% paraformaldehyde, cryoprotected (30% sucrose in PBS), and embedded in Tissue-Tek OCT compound and cryosectioned (8 μm). *In situ *hybridization using digoxygenin (Dig)-labeled probes was performed as described [75], with some modifications. Briefly, sections from E13.5 embryos were treated with 5 μg/ml proteinase K (Roche) in 0.1 M PBS for 15 minutes at room temperature prior to hybridization. The DIG signal was visualized with NBT/BCIP (Roche). The following probes were used: *Lhx2 *([GenBank:NM_010710], probe spanning the region 460–1750), *Nuak1 *([GenBank:NM_001004363], 2382–3143), *Tmem2 *([GenBank:BC076570], 296–1262), *Enc1 *([GenBank:NM_007930] 920–1793), and Etv5 ([GenBank:NM_023794], 661–1609).

## Authors' contributions

KR carried out generation and collection of material, confirmative analysis, in situ hybridizations and helped to draft the manuscript. LD carried out confirmative analysis, in situ hybridisations and helped to draft the manuscript. VW coordinated and carried out the manufacturing of the microarrays, carried out data analysis and statistical analysis of expression data and helped to draft the manuscript. SB performed the labelling and hybridisation procedures, participated in the analysis and helped to draft the manuscript. JL participated in the supervision and commented on the project, was responsible for funding at the KTH site. LC conceived of the study and helped to draft the manuscript. CW participated in the design of the study, its coordination, expression analyses and drafted the manuscript. All authors read and approved the final manuscript.

## Supplementary Material

Additional File 1 **Microarray expression data. **Values and annotations for genes differentially expressed at time points 36 h, 72 h and 96 h after dox withdrawal. Click here for file

Additional File 2 **Cell line expression data. **Differential expression between the cell lines DoxHPC1 and DoxHPC7 at time-point 0. Click here for file

Additional File 3 **Biological classification of data. **Complete listing of Gene Ontology classifications groups differentially expressed in this study. Click here for file

Additional File 4 **Overlap between genes defined as stem cell-specific in this study and HSC-specific datasets. **Listing of genes in our study overlapping with other studies of hematopoietic stem cells using Affymetrix technology (refs [29,30]). Click here for file

## Figures and Tables

**Table 1:** Number of array features and corresponding genes that are differentially expressed in the various comparisons. A false-discovery rate of 0.01 is used, with and without a M-value cut-off of 0.4.

	p < 0.01 (fdr)		p < 0.01 (fdr) and |M| > 0.4	
		
Comparison	probes	genes	probes	genes
A (36 vs. 0 h)	1718	1325	463	365
B (72 vs. 0 h)	2572	1936	695	525
C (96 vs. 0 h)	2562	1947	699	528
D (0 h vs. 0 h)	0	0	0	0
HPC1 vs. HPC7 (0 h)	2580	1995	646	498

**Table 2:** The top 25 down-regulated genes in the DoxHPC lines after dox withdrawal. The columns 36 h, 72 h and 96 h refer to hours after dox withdrawal and include the M-values (=log2 [expression in differentiated/expression in undifferentiated]). The M-value of *Lhx2 *down-regulation is equivalent to a fold-change of approximately 8.

Symbol	Gene name	Genbank	Unigene ID	Gene ID	36 h	72 h	96 h
*Lhx2*	LIM homeobox protein 2	CX208629	Mm.142856	16870	-2.95	-2.71	-3.06
*Serpina3g*	Serine (or cysteine) proteinase inhibitor, clade A, member 3G	CX234493	Mm.264709	20715	-2.02	-2.41	-2.53
*Bpgm*	2,3-bisphosphoglycerate mutase	CX200054	Mm.282863	12183	-1.63	-2.07	-2.06
*Csrp2*	Cysteine and glycine-rich protein 2	CX241650	Mm.2020	13008	-1.29	-1.28	-1.31
*Plscr1*	Phospholipid scramblase 1	CX230405	Mm.14627	22038	-1.21	-1.48	-1.64
*Mal*	Myelin and lymphocyte protein, T-cell differentiation protein	CX219564	Mm.39040	17153	-1.16	-1.14	-1.2
-	Unknown	CX220013	-	-	-1.13	-1.2	-1.1
*Sdh1*	Sorbitol dehydrogenase 1	CX204086	Mm.371580	20322	-1.1	-1	-1
*Upp1*	Uridine phosphorylase 1	CX218174	Mm.4610	22271	-1.09	-1.2	-1.33
*Syne2*	Synaptic nuclear envelope 2	CX218234	Mm.26652	319565	-1.02	-1.12	-1.12
*Catnd2*	DNA segment, KIST 4	CX237590	Mm.321648	18163	-1	-0.95	-1.02
*Galnt2*	UDP-N-acetyl-alpha-D-galactosamine: polypeptide N-acetylgalactosaminyl transferase 2	CX228653	Mm.33808	108148	-1	-1.1	-1.22
*Etv5*	Ets variant gene 5	CX240212	Mm.155708	104156	-0.99	-1.06	-1.16
*Myo1b*	Myosin IB	CX206461	Mm.3390	17912	-0.99	-1.17	-1.23
*Parp8*	RIKEN cDNA 5930433N17 gene	CX230711	Mm.274466	52552	-0.96	-0.83	-0.66
*Laptm4b*	Lysosomal-associated protein transmembrane 4B	CX240664	Mm.197518	114128	-0.92	-0.91	-0.95
*Nrgn*	Neurogranin	CX240939	Mm.335065	64011	-0.91	-0.9	-1.18
*B230104P22Rik*	RIKEN cDNA B230104P22 gene	CX241478	Mm.25874	77976	-0.89	-0.98	-0.99
*1810009M01Rik*	RIKEN cDNA 1810009M01 gene	CX239344	Mm.28385	65963	-0.89	-0.93	-1.07
*Sox4*	SRY-box containing gene 4	CX240226	Mm.240627	20677	-0.88	-1.04	-1.06
*2810408E11Rik*	RIKEN cDNA 2810408E11 gene	CX242247	Mm.291015	106200	-0.86	-0.88	-0.97
*Mfge8*	Milk fat globule-EGF factor 8 protein	CX239348	Mm.1451	17304	-0.86	-0.88	-1.01
*Serpina3n*	Serine (or cysteine) proteinase inhibitor, clade A, member 3N	CX200330	Mm.22650	20716	-0.86	-1.07	-1.15
*Vim*	Vimentin	CX235238	Mm.268000	22352	-0.85	-0.83	-0.81
*Cklfsf4*	Chemokine-like factor super family 4	CX206907	Mm.29658	97487	-0.85	-1.08	-1.16

**Table 3:** The top 25 up-regulated genes in the DoxHPC lines after dox withdrawal. The columns 36 h, 72 h and 96 h refer to hours after dox withdrawal and include the M-values (=log2 [expression in differentiated/expression in undifferentiated]).

Symbol	Gene name	Genbank	Unigene ID	Gene ID	36 h	72 h	96 h
*Slc2a3*	Solute carrier family 2 (facilitated glucose transporter), member 3	CX234108	Mm.269857	20527	2.04	1.5	1
*Bnip3*	BCL2/adenovirus E1B 19kDa-interacting protein 1, NIP3	CX229381	Mm.2159	12176	1.8	1.69	1.53
*Car2*	Carbonic anhydrase 2	CX241425	Mm.1186	12349	1.49	1.25	1.07
*Eno1*	Enolase 1, alpha non-neuron	CX240534	Mm.70666	13806	1.39	1.27	1.15
*Aldoa*	Aldolase 1, A isoform	CX237582	Mm.275831	11674	1.37	1.52	1.27
*2410129H14Rik*	RIKEN cDNA 2410129H14 gene	CX231735	Mm.38912	76789	1.36	1.19	1.05
*Ndrg1*	N-myc downstream regulated gene 1	CX218239	Mm.30837	17988	1.35	0.86	0.71
*Ncf1*	Neutrophil cytosolic factor 1	CX225706	Mm.4149	17969	1.3	1.48	1.62
-	Unknown	CX635138	-		1.29	1.49	1.64
*Perp*	PERP, TP53 apoptosis effector	CX231659	Mm.28209	64058	1.29	1.29	1.08
*Pgk1*	Phosphoglycerate kinase 1	CX242304	Mm.336205	18655	1.27	1.32	1.22
-	Unknown	CX207798	-	-	1.19	1.16	1.1
-	Unknown	CX241476	-	-	1.18	1.08	1.05
-	Unknown	CX200988	-	-	1.18	1.09	1.06
*Tpi1*	Triosephosphate isomerase 1	CX242931	Mm.4222	21991	1.17	1.07	1.03
	Unknown	CX207886	-		1.15	0.98	0.84
*Mcm10*	Minichromosome maintenance deficient 10 (S. cerevisiae)	CX214265	Mm.23828	70024	1.15	1.34	1.2
*Txnip*	Thioredoxin interacting protein	CX201093	Mm.271877	56338	1.14	1.32	1.15
*P4ha1*	Procollagen-proline, 2-oxoglutarate 4-dioxygenase (proline 4-hydroxylase), alpha 1 polypeptide	CX231610	Mm.2212	18451	1.13	0.82	0.81
*2610034M16Rik*	RIKEN cDNA 2610034M16 gene	CX201694	Mm.278512	69239	1.11	1.31	1.26
*9830134C10Rik*	RIKEN cDNA 9830134C10 gene	CX233114	Mm.328927	442827	1.1	1.43	1.37
*2610034H20Rik*	RIKEN cDNA 2610034H20 gene	CX225894	Mm.250541	76375	1.09	1.05	0.99
*Ldh1*	Lactate dehydrogenase 1, A chain	CX239308	Mm.29324	16828	1.08	0.92	0.83
*A830010M20Rik*	RIKEN cDNA A830010M20 gene	CX237601	Mm.261891	231570	1.07	1.19	1.02
*Tec*	Cytoplasmic tyrosine kinase, Dscr28C related (Drosophila)	CX228815	Mm.319581	21682	1.07	1	0.97

**Table 4:** The most regulated groups genes. Enriched biological processes (level 3), molecular function and chromosome distribution as defined by Gene Ontology and pathways as defined by Kegg of the differentially expressed genes.

Categorial system	Differential Expression	Term	Genes	p-value
Biological processes	Down-regulated	REGULATION OF SIGNAL TRANSDUCTION	4	0.015
		ORGANOGENESIS	10	0.070
		CELL DEATH	6	0.077
	Up-regulated	ALCOHOL METABOLISM	16	0.00000000000087
		MACROMOLECULE METABOLISM	38	0.0000080
		BIOSYNTHESIS	19	0.000086
		CATABOLISM	16	0.00064
		COENZYME AND PROSTHETIC GROUP METABOLISM	6	0.0028
		VITAMIN METABOLISM	3	0.035
		AMINE METABOLISM	5	0.063
Molecular function	Down-regulated	TRANSFERASE ACTIVITY, TRANSFERRING GLYCOSYL GROUPS	6	0.013
		OXIDOREDUCTASE ACTIVITY, ACTING ON PEROXIDE AS ACCEPTOR	3	0.030
		PROTEASE INHIBITOR ACTIVITY	4	0.087
	Up-regulated	CARBOHYDRATE KINASE ACTIVITY	3	0.0096
		TRANSFERASE ACTIVITY, TRANSFERRING PHOSPHORUS-CONTAINING GROUPS	12	0.013
		CARBON-OXYGEN LYASE ACTIVITY	3	0.054
		PURINE NUCLEOTIDE BINDING	15	0.061
Chromosome	Down-regulated	2	17	0.053
	Up-regulated	8	12	0.021
Kegg pathways	Up-regulated	PENTOSE PHOSPHATE PATHWAY	6	0.0000029
		GLYCOLYSIS / GLUCONEOGENESIS	10	0.00011
		GALACTOSE METABOLISM	5	0.00018
		FRUCTOSE AND MANNOSE METABOLISM	5	0.00061
		CARBON FIXATION	4	0.0018
		STARCH AND SUCROSE METABOLISM	4	0.026
